# American Medical Association

**Published:** 1884-05-20

**Authors:** 


### *AMERICAN MEDICAL ASSOCIATION.*

The American Medical Association convened in Washington City on the 6th inst., and was largely attended, there being about five hundred members present. Dr. Garnett made the address of welcome, and the President, Dr. Austin Flint, delivered his annual address. He said much on the great progress of medical science. Among the suggestions in the address was one advising the appointment of a committee to confer with the Faculties of medical schools with a view to secure uniformity in the *requirements* for graduation of students.

In regard to refusing to consult with irregular practitioners, he advised that the Association should specify more particularly the reasons therefor. He made, in the conclusion of his address, a feeling tribute to Dr. S. D. Gross, of Philadelphia.

Officers for ensuing year:

*President*—Henry F. Campbell, Georgia.

*Vice-Presidents*—J. S. Lynch, Maryland; S. D. Mercer, Nebraska; J. W. Parsons, New Hampshire; H. C. Ghent, Texas.

*Treasurer*—R. J. Dunglison, Pennsylvania.

*Librarian*—C. H. Kleinschmidt, Disirict of Columbia.

*Chairman of Committee of Arrangements*—S. D. Logan, New Orleans.

*Assistant Secretary*- W. H. Watkins, New Orleans.

Place of meeting, New Orleans, last Tuesday in April, 1885.

